# Metabolic protein kinase signalling in neuroblastoma

**DOI:** 10.1016/j.molmet.2023.101771

**Published:** 2023-07-04

**Authors:** William J. Smiles, Luca Catalano, Victoria E. Stefan, Daniela D. Weber, Barbara Kofler

**Affiliations:** Research Program for Receptor Biochemistry and Tumor Metabolism, Department of Pediatrics, University Hospital of the Paracelsus Medical University, Müllner Hauptstraße 48, 5020, Salzburg, Austria

**Keywords:** Neuroblastoma, Metabolism, Kinase, Signalling

## Abstract

**Background:**

Neuroblastoma is a paediatric malignancy of incredibly complex aetiology. Oncogenic protein kinase signalling in neuroblastoma has conventionally focussed on transduction through the well-characterised PI3K/Akt and MAPK pathways, in which the latter has been implicated in treatment resistance. The discovery of the receptor tyrosine kinase ALK as a target of genetic alterations in cases of familial and sporadic neuroblastoma, was a breakthrough in the understanding of the complex genetic heterogeneity of neuroblastoma. However, despite progress in the development of small-molecule inhibitors of ALK, treatment resistance frequently arises and appears to be a feature of the disease. Moreover, since the identification of ALK, several additional protein kinases, including the PIM and Aurora kinases, have emerged not only as drivers of the disease phenotype, but also as promising druggable targets. This is particularly the case for Aurora-A, given its intimate engagement with MYCN, a driver oncogene of aggressive neuroblastoma previously considered ‘undruggable.’

**Scope of review:**

Aided by significant advances in structural biology and a broader understanding of the mechanisms of protein kinase function and regulation, we comprehensively outline the role of protein kinase signalling, emphasising ALK, PIM and Aurora in neuroblastoma, their respective metabolic outputs, and broader implications for targeted therapies.

**Major conclusions:**

Despite massively divergent regulatory mechanisms, ALK, PIM and Aurora kinases all obtain significant roles in cellular glycolytic and mitochondrial metabolism and neuroblastoma progression, and in several instances are implicated in treatment resistance. While metabolism of neuroblastoma tends to display hallmarks of the glycolytic “Warburg effect,” aggressive, in particular *MYCN*-amplified tumours, retain functional mitochondrial metabolism, allowing for survival and proliferation under nutrient stress. Future strategies employing specific kinase inhibitors as part of the treatment regimen should consider combinatorial attempts at interfering with tumour metabolism, either through metabolic pathway inhibitors, or by dietary means, with a view to abolish metabolic flexibility that endows cancerous cells with a survival advantage.

## Highlights

• Neuroblastoma is characterised by genetic and metabolic heterogeneity depending on disease stage/severity.
• *MYCN*-amplification is a feature of high-risk disease.
• Neuroblastoma metabolism displays both glycolytic features and functional mitochondrial respiration.
• Divergent protein kinases ALK, PIM and Aurora are implicated in neuroblastoma progression and treatment resistance.
• These kinases are metabolic regulators, thus explaining their oncogenicity and attractiveness as therapeutic targets.

## 1 Introduction

Neuroblastoma is the second most common extra-cranial solid malignancy in children and the most common solid tumour of infancy, accounting for approximately 15% of paediatric cancer deaths [1]. Neuroblastomas originate from precursor cells of the sympathetic/peripheral nervous system, typically forming within paraspinal locations such as the abdomen or chest [2]. The average age at diagnosis of patients with familial neuroblastoma is 9 months, as opposed to the average of 18 months observed in the general population [2]. Neuroblastoma has been reported in infant twins, pointing to hereditary factors, which is in contrast to the discordance of disease in older twins, suggesting spontaneous mutations [3]. The most common chromosomal aberrations in neuroblastoma are allelic loss of 1p36 and 11q, and gain of 17q [[4], [5], [6], [7], [8]]. Loss of the short arm of chromosome 1 (1p), in particular, frequently coincides with amplification of *MYCN* on chromosome 2p24. *MYCN*-amplification is a major hallmark of disease with a 20–25% incidence rate that can increase to 40% in high-risk scenarios, associating with poor prognosis [9,10]. Some of the earliest evidence that MYCN contributes to neuroblastoma was demonstrated in transgenic mice overexpressing MYCN in neuroectodermal cells [11]. These mice developed neuroblastoma with recurrent chromosomal copy number abnormalities, highlighting that genetic mutations coinciding with MYCN overexpression are involved in the transformation of neuroblasts and disease genesis [11]. Roughly half of patients present to the clinic showing signs of metastasis at diagnosis [12], with bone, lymph nodes and the liver being the major sites of metastatic dissemination [13]. *MYCN*-amplification correlates with neuroblastoma metastasis [[14], [15], [16]], by contributing to a number of processes implicated in cell migration and invasion [17]. Unfortunately, MYCN is generally considered undruggable due to the existence of two extended α-helices in the DNA-binding domain devoid of surfaces suitable for small-molecule interactions. Compounding this challenge is the fact that MYCN has no enzymatic function, as a monomer its N-terminal transactivating domain is intrinsically disordered, and MYCN is highly homologous to other MYC family members (e.g., c-Myc), for whom off-target inhibition could disrupt vital cellular processes in non-cancerous cells [18]. However, as this review will point out, the discovery of novel, MYCN-interacting proteins overexpressed in neuroblastoma, such as Aurora kinase A, provide exciting opportunities for pharmacological strategies aimed at downregulating MYCN independent of its transcriptional function. The closely related *c-MYC* is also a potent transforming gene in a subset of high-risk neuroblastoma cases (∼10%) and feature of malignant progression in stage four non-*MYCN*-amplified tumours [19,20].

Other notable genetic changes in neuroblastoma include overexpression of ornithine decarboxylase 1 (ODC1), the rate-limiting enzyme in polyamine biosynthesis, activating mutations in the receptor tyrosine kinase (RTK) anaplastic lymphoma kinase (ALK), and loss-of-function mutations in the homeobox gene *PHOX2B* that are present in children that develop sporadic or familial neuroblastoma [[21], [22], [23]]. Unlike numerous other cancers, mutations in the tumour suppressor p53 are rare at diagnosis in neuroblastoma, despite abnormalities in the p53 pathway occurring in close to 50% of relapsed neuroblastoma and contributing to chemotherapy resistance [24]. *MYCN*-amplification-induced overexpression of the ubiquitin ligase mouse double minute 2 (MDM2), that targets p53 for degradation, is one proposed mechanism contributing to this treatment resistance [24,25], while in *MYCN*-wild-type tumours, overexpression of the epigenetic regulator and histone methyltransferase SETD8, that also methylates and inactivates p53, was linked to high-risk neuroblastoma [26]. Finally, a S120G mutation of NDPK-A, a nucleoside-diphosphate kinase that produces ATP, has been associated with advanced neuroblastoma and metastasis [27,28].

## 2 Metabolism of neuroblastoma

Neuroblastoma tumours have high-glucose uptake [29,30], which at first glance is predictive of the Warburg effect of elevated rates of glycolysis and low mitochondrial respiration despite the presence of oxygen. This was confirmed in neuroblastoma cell lines that exhibited elevated lactate production commensurate with low oxygen consumption and a reduction in cell viability when treated with the glycolysis inhibitor 3-BrOP [31]. However, these metabolic effects are despite appreciable mitochondrial content found in neuroblastoma [32], possibly explaining, in part, the metabolic flexibility of some neuroblastoma cell lines that switch to oxidative metabolism when glucose availability is limited [33,34]. Nevertheless, when compared to healthy tissue, human neuroblastoma specimens have a marked reduction in mitochondrial DNA (mtDNA) content and oxidative phosphorylation (OXPHOS) enzyme activity, particularly at the level of complex II [35,36]. Defective complex II activity is unlikely to be a consequence of mutations to, or epigenetic inactivation of, complex II subunit-encoding genes [37,38], despite the complex II gene *SDHB* mapping to chromosome 1p36, a region of frequent loss of heterozygosity in neuroblastoma. Low mtDNA copy number in neuroblastoma appears to be a feature of other glycolytic cell types (e.g., neural stem cells, glioblastoma cells) and may contribute to their poor differentiation propensity [39,40]. At the point of differentiation, neural stem cells upregulate mtDNA copy number and respiratory capacity [39], suggesting this might be a characteristic of the malignant phenotype. Gain of chromosome 17q in high-stage neuroblastoma is associated with overexpression of the anti-apoptotic and mitotic protein Survivin [41]. In high-stage neuroblastoma cells, Survivin induces mitochondrial fragmentation (‘fission’) via the GTPase dynamin-related protein 1 (Drp1) that produces two daughter organelles that either fuse with the existing mitochondrial network or are degraded via autophagy [42]. This mitochondrial fission event reduces respiration by inhibiting complex I, attenuating reactive oxygen species (ROS) production that left unchecked, instigates the cell death cascade. Accordingly, to compensate for the absence of energy generated by mitochondrial respiration, these cells upregulate glycolysis [42].

*MYCN*-amplification leads to upregulation of hypoxia-inducible factor-1α (HIF-1α), a transcription factor that in concert with MYCN, enhances the expression of glycolytic genes and drives neuroblastoma tumour progression [43]. However, in three neuroblastoma cell lines of varying MYCN status, there was no association between MYCN expression and the Warburg effect [44], in which the BE (2)-C cell line of the highest MYCN content satisfied the bulk of their energy demands through mitochondrial respiration. In fact, *MYCN*-amplification has been shown to promote both glycolysis *and* OXPHOS in neuroblastoma [45], and its inhibition triggers a mitochondrial respiratory chain defect and accumulation of lipid droplets, signifying a reduction in fatty acid oxidation [46]. Hence, despite some signs indicating a reliance on glycolytic metabolism, there is a retention of mitochondrial function required for the growth and survival of neuroblastoma, whereby MYCN seems to occupy a position of prominence in controlling both of these major metabolic pathways. *MYCN*-amplification in neuroblastoma elevates fatty acid uptake, exposing a metabolic vulnerability that can be exploited by targeting FATP2, a fatty acid transport protein regulated by MYCN whose inhibition supresses tumour growth *in vivo* [47]. In addition, deprivation of the anaplerotic amino acid glutamine in *MYCN*-amplified neuroblastoma cells, triggers apoptosis [48], highlighting a glutamine addiction necessary to replenish tricarboxylic acid (TCA) cycle intermediates that sustain cell viability, and consistent with the finding of augmented *de novo* glutamine synthesis in *MYCN*-amplified neuroblastoma cells [45]. It was also reported that *MYCN*-amplified neuroblastoma cells rely on the amino acid transporter ASCT2 (solute carrier family 1 member 5, SLC1A5) for the provision of glutamine, in which its expression correlates with poor patient survival [49]. Moreover, loss of dihydrolipoamide S-succinyltransferase (DLST) that modulates entry of glutamine into the TCA cycle, impeded progression of *MYCN*-amplified neuroblastoma by impairing NADH production and OXPHOS activity [50]. Elevated *DLST* expression among high-risk neuroblastoma patients correlated with an enrichment of genes within the OXPHOS network, while OXPHOS inhibition perturbed the aggressive properties of *MYCN*-amplified neuroblastoma xenografts [50]. Finally, in addition to glutamine, MYCN also controls cysteine addiction in neuroblastoma [51]. Overexpression of MYCN sensitises neuroblastoma cells to redox stress and lipid peroxidation upon cysteine limitation, triggering ferroptotic (iron-dependent) cell death, and exposing another metabolic liability in these tumours that was capitalised on by targeting cysteine import and/or metabolism [51]. Despite the heterogeneity of neuroblastoma metabolism in general, there appears to be a specific pattern in high-risk, *MYCN*-amplified disease involving a metabolic transformation favouring fatty acid uptake and mitochondrial respiration and preferential utilisation of glutamine and cysteine for cell survival, that altogether uncover novel opportunities for targeted therapeutics.

## 3 Canonical protein kinase signalling in neuroblastoma

**Figure 1:**
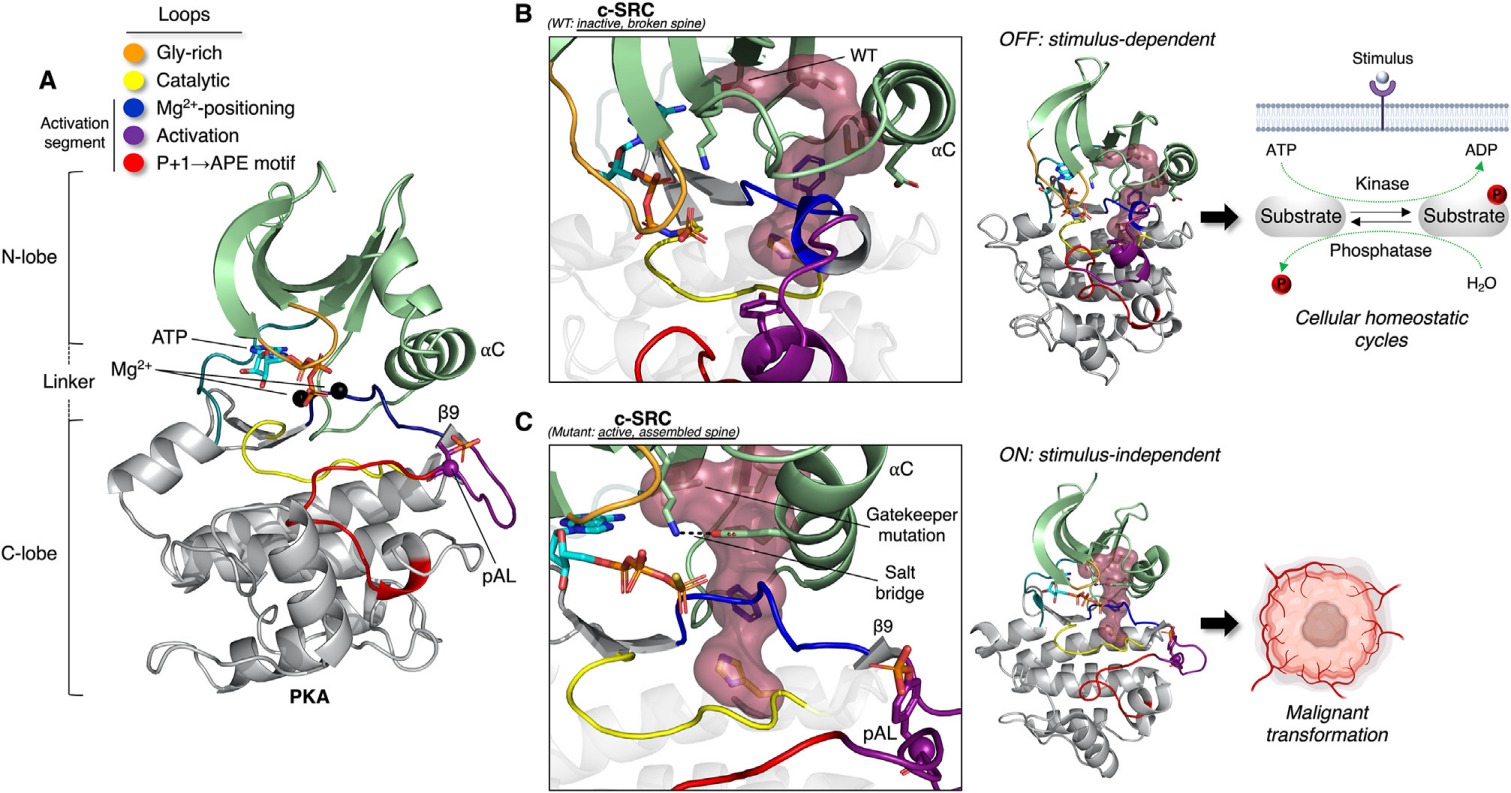
Typical architecture of a eukaryotic protein kinase (ePK) and structural considerations in cancer. **A**) PKA (PDB: 1ATP) is displayed as the prototypical model of an ePK. The catalytic core is defined by N- and C-terminal lobes separated by a linker that creates an active site cleft accommodating ATP and two divalent cations (Mg^2+^). The N-lobe consists of a five-stranded β-sheet and mobile αC helix (note, the preceding αB helix is not always present in ePKs), while the C-lobe is predominantly helical, typically α-helices D through to I. The major conserved loops and motifs, from N- to C-termini, are indicated in the colour-coded key and this colour scheme remains consistent throughout the manuscript. Briefly, the glycine (Gly)-rich loop connecting the first two β-strands of the N-lobe helps position ATP for catalysis. All subsequent regions of interest are located in the C-lobe. The catalytic loop contains a critical HRD (His-Arg-Asp) motif involved in phospho-transfer and stabilisation of the activation loop. The DFG motif is also referred to as the magnesium-positioning loop. A feature of an active kinase is also a small, β9-strand formed between the DFG motif and activation loop. As touched upon, the activation loop contains the important phosphorylation site (‘pAL’ in the figure) that is a signature of most active protein kinases (e.g., the basic HRD motif arginine interacts with the negatively charged phosphate group). Lastly, the P+1 loop and APE motif generally help position a substrate during the catalytic cycle. **B**) Several residues from these loops contribute to an internal regulatory ‘spine’ that is disrupted in the inactive kinase (presented as surface representation). In the case of the tyrosine kinase c-SRC (PDB: 2SRC), this is due to a threonine residue occupying the so-called gate-keeper position in the 5th β-stand of the N-lobe, ensuring fidelity of phospho-turnover cycles inherent to cellular homeostasis. **C**) Substitution of this residue to a bulkier hydrophobic amino acid (in this case isoleucine; PDB: 3DQW) results in constitutive spine formation and kinase activation, as evidenced by several other features including, but not limited to, pAL, formation of the β9 strand, and a salt bridge between an αC helix glutamate with a lysine in the β3-strand. Without the normal homeostatic restraints placed on activating an ePK, this single mutation is sufficient to trigger malignant transformation.

Unlike enzymes in metabolic pathways that are commissioned to turnover large amounts of substrate, protein kinases have evolved to specifically function as molecular on/off “switches” and not efficient catalysts, largely by virtue of their internal architecture that can dynamically assemble and disassemble in response to physiological stimuli [52]. Structurally, protein kinases are bilobal, consisting of N- and C-terminal lobes separated by a hinge/linker that creates an ATP-accommodating cleft and site of phospho-transfer toward a peptide substrate. The fundamental structure and major regulatory elements of a protein kinase, using protein kinase A (PKA) as a template, is presented in Figure 1A (additional details are provided in the legend), including the activation loop phosphorylation site that is typically either targeted by an alternate upstream kinase or by a *trans* mechanism, involving transient dimerization and reciprocal phosphorylation of the opposing molecule. The importance of this on/off switch-like function, in the context of cancer, is perhaps best illustrated by the resistance to small-molecule inhibitors caused by mutation of a gatekeeper threonine to a bulkier hydrophobic residue in the active site of the c-Abl, c-SRC, PDGFR and EGFR protein kinases [53]. This single mutation stabilises the internal architecture of the molecule, generating a regulatory ‘spine’ that is absent in the inactive, wild-type kinase, thereby endowing the enzyme with constitutive activity sufficient to trigger malignant transformation of mammalian cells (structural comparisons of c-SRC as an example is provided in Figure 1B,C) [53].

Protein kinase signalling research in neuroblastoma has traditionally centred on the phosphatidylinositol-3 kinase (PI3K)/Akt and Ras/mitogen-activated protein kinase (MAPK) pathways, fundamental regulators of cell metabolism, growth, proliferation and survival, and two of the most commonly studied signalling pathways in human cancers [54,55]. Both pathways converge on and activate the mammalian target of rapamycin (mTOR) protein kinase in complex 1 (mTORC1), a major nutrient sensor and driver of cellular anabolism [56]. Akt activation, as denoted by an activatory phosphorylation status, has been linked to advanced disease in neuroblastoma, correlating with *MYCN*-amplification and poor prognosis [57]. Canonically, Akt is switched on by growth factors and cytokines, in which the class Ia PI3K is scaffolded to a RTK and catalyses plasma membrane phosphatidylinositol-3,4,5-trisphosphate (PIP_3_) formation that serves as a docking site for Akt via its pleckstrin homology domain and relieves basal kinase autoinhibition [58,59]. Akt is phosphorylated on its hydrophobic motif S473 by mTOR in a second complex, mTORC2, which has distinct functions and binding partners to that of mTORC1. This promotes association with the master regulatory kinase, phosphoinositide-dependent protein kinase 1 (PDK1) that fully activates Akt by phosphorylation on the T308 activation loop residue [60]. Alternatively, a positive feedback loop has been elucidated in which PDK1-activated Akt phosphorylates T86 of the mTORC2 component SIN1, activating the complex which then phosphorylates Akt on S473 [61]. Regardless, both of these Akt phosphorylation sites have been associated with poor prognosis in neuroblastoma [57]. The Akt substrate glycogen synthase kinase 3β (GSK3β) regulates MYCN stability (discussed in greater detail in Section 6.1. on Aurora-A) [62], and this appears to be particularly reliant on mTORC2 inhibition and relief of S473 phosphorylation of Akt [63]. As a side note, mTORC2, downstream of PI3K, was previously found to control the expression of HIF-2α in neuroblastoma cells isolated from metastatic patient-derived xenografts, inducing vascularisation *in vivo* [64]. The PI3K/Akt signal is ultimately terminated by negative feedback transmitted by mTORC1 (e.g., inhibitory serine phosphorylation of the insulin receptor substrate 1) and via the tumour suppressor, phosphatase and tensin homologue (PTEN), a lipid phosphatase for PIP_3_ that removes the Akt membrane-binding stimulus [54]. Genetic aberrations causing constitutive activation of PI3K/Akt signalling is commonplace in numerous cancers. These include activation mutations in *PIK3CA* (encoding the PI3K catalytic subunit), loss-of-function mutations in or deletions to *PTEN*, RTK amplification, and amplification and gain-of-function mutations in genes encoding Akt (which exists as three isoforms) [54,65]. However, there are no known reports of genetic changes to *Akt* in neuroblastoma, while *PTEN* deletions have been shown to contribute to the progression of only a very small proportion of human neuroblastoma cell lines and primary tumours [66]. ALK is an activator of Akt signalling and is implicated in a positive feedback loop with MYCN, which may reconcile the aforementioned association between Akt signalling and *MYCN*-amplification in the absence of direct genetic alterations to the PI3K/Akt pathway.

MAPK signalling involves a three-tiered phosphorylation cascade comprising the effector MAPK, a MAPK kinase (MAPKK), and a MAPKK kinase (MAPKKK). The Ser/Thr extracellular signal-regulated kinases 1 and 2 (ERK1/2, 83% homology) are well-known members of the MAPK family (MAPK3 and MAPK1, respectively) and are activated in response to growth factors (e.g., EGF, PDGF), phorbol esters and cytokines [67]. The first two tiers of this pathway are comprised of either A-Raf, B-Raf, or Raf-1 (also referred to as C-Raf) functioning as the MAPKKK, then MEK1/2 as the MAPKK. In response to an extracellular cue, ligand binding to a RTK, then dimerization and autophosphorylation, creates docking sites for phospho-tyrosine-binding proteins such as growth factor receptor-bound protein 2 (Grb2) that in turn, recruit the guanine nucleotide exchange factor son of sevenless (SOS). SOS then catalyses GTP loading and activation of the Ras GTPase H-, K- or N-Ras, activating the respective MAPKKK following its plasma membrane recruitment to kick-start the phosphorylation cascade [68].

Genetic alterations to the Ras/MAPK pathway are frequently observed in relapsed neuroblastoma following chemotherapy [69]. A number of these mutations have been found in the upstream ALK receptor, which transmits signals through this pathway. As we will point out in this review, resistance to pharmacological inhibition of ALK not only implicates activation of Ras/MAPK signalling, but the opposite is also apparent; hence, MAPK inhibitors provoke ALK signalling to Akt. Moreover, resistance to ALK inhibitors uncovered a novel oncogenic driver of neuroblastoma, Provirus Integration Site for Moloney Murine Leukemia Virus (PIM) kinase. Therefore, we will focus on the roles of ALK and PIM in neuroblastoma, their critical signalling nodes, and regulation of cellular metabolism. We will also discuss the emergence of the Aurora-A and -B protein kinases, their unique contribution to the aggressiveness of *MYCN*-amplified tumours, and underappreciated regulation of cellular bioenergetics.

## 4 Anaplastic lymphoma kinase (ALK)

ALK is a member of the insulin receptor family and bears high-sequence similarity with the leukocyte tyrosine kinase (LTK) RTK [70]. Discovered in 1994 in non-Hodgkin's lymphoma as a result of a t (2; 5) (p23:q35) chromosomal translocation event, ALK was found to be expressed as a chimaeric protein, in which its kinase domain was fused to the N-terminal region of the nucleolar protein nucleophosmin [71,72]. This fusion event creates a dimerization domain for ALK, resulting in a constitutively active protein with oncogenic potential. In fact, numerous, alternate fusion partners have been documented following the discovery of ALK translocations, for example EML4-ALK-positive non-small-cell lung cancer (NSCLC) and other solid tumours [73,74].

**Figure 2:**
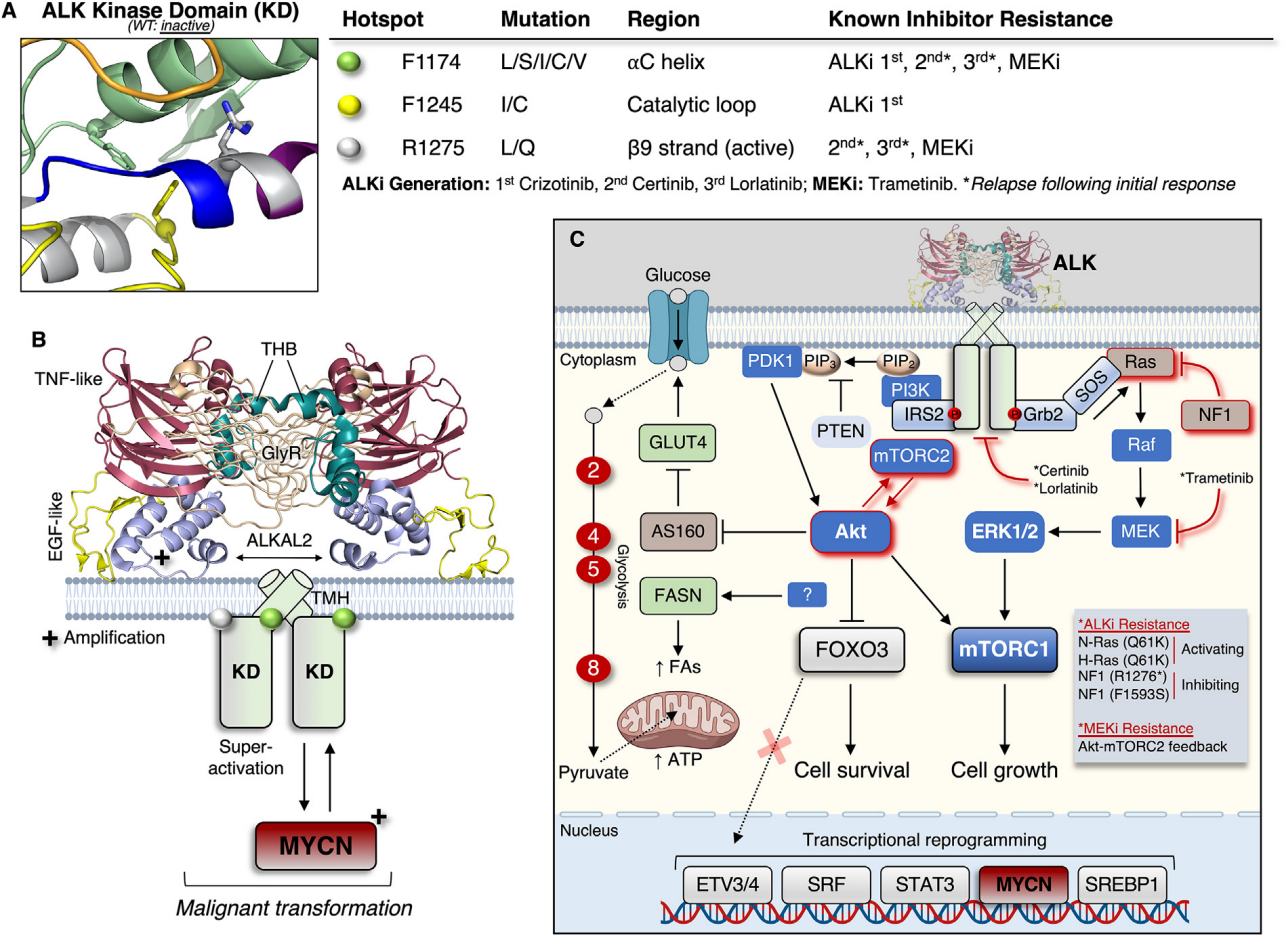
ALK drug resistance and signalling in neuroblastoma. **A**) Three kinase domain ‘hotspot’ mutations have been identified (indicated by coloured spheres in the structure of the inactive, unliganded ALK kinase domain (PDB: 3L9P)) in neuroblastoma that are responsible for, or associated with, resistance to ALK or MEK inhibitors, resulting in MAPK and Akt signalling, respectively. An asterisk refers to cases in which ALK-mutated tumours develop resistance (e.g., N-Ras Q61K) following a period of initial positive responsiveness to the drug. Note that the R1275L/Q mutation is located where a β9-strand would normally form in the active kinase, as can be seen in Figure 1A. **B**) Structure (PDB: 7N00) of the dimeric, ligand-bound extracellular domain of ALK showcases several novel features of RTK including a tri-helical bundle (THB), and the glycine-rich (GlyR) and TNF- and EGF-like domains. A transmembrane helix (TMH) connects the extracellular domain to the intracellular kinase domain. In this case the more potent ALKAL2 is shown in the structure, although it should be noted that ALKAL1 is also an activatory ligand. *ALKAL*- and *MYCN*-amplification (+) are generally mutually exclusive, and ALKAL activation of mutant ALK generates a so-called super-active kinase that, in addition to mutant ALK (F1174L) alongside *MYCN* overexpression, promotes malignant transformation. **C**) Major ALK downstream signalling effectors and transcriptional modulators, and the metabolic consequences of ALK activity, including putative phospho-activation of FASN and upregulation of several enzymes in the glycolytic pathway that are numbered in red circles according to order (2: glucose-6-phosphate isomerase; 4: aldolase; 5: triosephosphate isomerase 1; 8: phosphoglycerate mutase 1). ALK and MEK drug resistance mechanisms, by way of signalling pathway activation, are indicated by red lines and described in the schematic text box.

Full-length ALK is expressed as a 177 kDa protein that undergoes N-terminal glycosylation to yield a final molecular mass of ∼220 kDa [70,75]. The mature protein displays expected features of a RTK (e.g., extracellular ligand binding and transmembrane domain) [75], although the overall extracellular architecture is atypical. This is by virtue of a glycine-rich region (GlyR), TNF- and EGF-like domains, as well as a tri-α-helical bundle important for dimerization and activation upon ligand binding (domain architecture of published coordinates (PDB: 7N00) presented in Figure 2B) [76,77]. The secretory cytokines ALKAL1 (FAM150A/Augmentor-β) and ALKAL2 (FAM150B/Augmentor-α) are physiological ligands for both ALK and LTK, whereby ALKAL2 has the greatest affinity toward ALK [78]. Once ligand-bound, ALK activation takes place in a canonical manner involving sequential autophosphorylation reactions of three kinase domain activation loop tyrosine residues (Y1278 is initially phosphorylated, followed by phosphorylation of Y1282 and Y1283 [79]), yet alternate and/or cooperative mechanisms of activation (i.e., other phosphorylation events) remain possible [80].

In humans, ALK is expressed in the small intestine, testis, and brain, but not in normal lymphoid cells [72]. Consistent with its prominence in the brain, mice lacking *ALK* display neurochemical (increased basal dopaminergic signalling within the frontal cortex) [81] and behavioural alterations (e.g., increased alcohol consumption) [82], leading researchers to flag it as a potential therapeutic target for psychiatric disorders such as schizophrenia and depression [81]. Moreover, loss of *ALK* protects mice from diet- and leptin (mutation)-induced obesity in a manner involving its hypothalamic control of energy expenditure via adipose tissue lipolysis [83]. As such, genetic (intronic) variants in *ALK* have been associated with ‘thinness’ in metabolically healthy humans and *Drosophila*, demonstrating a conserved metabolic role [83].

### 4.1 Role of ALK in neuroblastoma

Full-length ALK is expressed in neuroblastoma [84], and is a fairly frequent target of genetic alterations leading to activating mutations in cases of both familial (1–2%) and sporadic (6–10%) primary neuroblastoma [[85], [86], [87], [88]]. Mutations to F1174 (L/S/I/C/V substitutions), F1245 (I/C), and R1275 (L/Q) in the kinase domain are three hotspots (Figure 2A) observed in ∼90% of mutated cases of neuroblastoma, for which the most frequent, F1174L and R1275Q, markedly enhance ALK autophosphorylation and activation [89]. *ALK* is a direct transcriptional target of MYCN [90], and through positive feedback ALK can upregulate *MYCN* gene transcription itself [91]. *ALK* amplification is also observed in approximately 15% of *MYCN*-amplified primary neuroblastomas, associating with worsened clinical outcomes [86,87]. The F1174L mutation is particularly oncogenic, as it augments lethality when accompanied by *MYCN* overexpression (Figure 2B) [92,93]. For example, the collaborative actions of ALK and MYCN provide a survival advantage to hyperplastic neuroblasts [92]. The ALK ligand, ALKAL2, has higher expression in non-*MYCN*- versus *MYCN*-amplified tumours [94], although it is still capable of enhancing MYCN-driven neuroblastoma even in the absence of hotspot ALK mutations [95]. The tumourigenic potential of ALKAL2 is however, highest when interacting with mutant (F1174L, R1275Q) ALK, forming a ‘super-active’ kinase complex (Figure 2B) [96]. ALK containing truncated extracellular domains triggering constitutive kinase activity have also been described in neuroblastoma [97,98], as well as a novel, recently discovered ALK fusion chimera with the teneurin transmembrane protein 3 (TENM3), arising from translocation within chromosome 2p and 4q that causes oncogenic transformation [99].

ALK cell signalling has been studied predominantly in the context of the oncogenic effects of expressed fusion proteins in cancers other than neuroblastoma (e.g., NSCLC). These effects have been reviewed extensively [73,80] and is not within the scope of the present article. In neuroblastoma, several studies combining high-throughput proteomics and RNA-sequencing techniques identified ERK1/2 and Akt as major downstream effector kinases of full-length ALK. Here, their activation culminated in upregulation of gene expression networks via phosphorylation of several transcription factors, including the forkhead box member FOXO3, SRF, STAT3, and members of the ETS family (ETV3/4) (whose expression levels correlated with poor disease prognosis) (Figure 2C) [[100], [101], [102]]. For example, ALK promoted the Akt-mediated phosphorylation and cytoplasmic retention of FOXO3 to ensure cell survival in a manner implicating engagement of the insulin receptor substrate 2 (IRS2) [100,101], which is best regarded for its role in conventional growth factor signalling. As previously mentioned, both ERK1/2 and Akt converge to activate mTORC1, making it hardly surprising that ALK activation in neuroblastoma cells upregulates mTORC1 [95].

Five ALK inhibitors have been approved for the treatment of ALK-positive NSCLC. The first-in-class ALK inhibitor crizotinib, limited the growth of neuroblastoma cell lines expressing amplified wild-type and R1275Q-mutated ALK, but not F1174L-mutated ALK [103]. The F1174L mutation was later found to be a mechanism of treatment resistance in neuroblastoma [104,105]. In preclinical xenograft models, this treatment resistance could be overcome by the addition of chemotherapy, provided p53 expression remained intact; in other words, the beneficial effect of chemotherapy combined with ALK inhibition was abrogated by loss of p53 [106]. Phase I clinical trials using the second-generation ALK inhibitor ceritinib demonstrated responsiveness in a subset of patients with refractory or relapsed neuroblastoma, with indications that individuals bearing the R1275Q mutation were more sensitive to treatment [107]. The third-generation ALK inhibitor lorlatinib overcomes crizotinib resistance *in vivo* in patient-derived xenografts harbouring F1174L or F1245C mutations [108,109], and is presently being investigated in high-risk neuroblastoma patients (clinical trial: NCT03126916) following promising early reports of anti-tumour activity and tolerability in a phase I clinical trial [110]. In a clinical case study of a relapsed, metastatic ALK F1174L-mutated neuroblastoma patient, there was a complete response to lorlatinib initially that relapsed after 13 months, associating with development of an activating N-Ras Q61K mutation [111]. This finding was independently corroborated in tumour specimens from two lorlatinib-treated patients, while an additional two ceritinib-treated patients displayed *de novo* loss-of-function mutations in the gene encoding neurofibromin (*NF1*; R1276∗ nonsense, F1593S) [112]. NF1 is a GTPase activating protein and negative regulator of Ras/MAPK signalling and its dysfunction results in constitutive activation of the latter pathway manifesting as drug resistance. Lorlatinib efficacy may be potentiated by the inclusion of MDM2 inhibitors to preserve p53 function and address chemoresistance [113]. Lastly, proliferation of ALK-addicted neuroblastoma cell lines (expressing either amplified or hotspot-mutated ALK) was found to occur independent of MAPK signalling, whereby exposure to the MEK inhibitor trametinib upregulated the positive feedback loop between Akt and mTORC2 [114]. Tumour growth was stunted by trametinib in N-Ras mutant (Q61K) neuroblastoma and EML4-ALK fusion-positive NSCLC xenografts, but not in the ALK-addicted neuroblastoma tumours [114]. Given their complexity, we have summarised and schematically depicted these drug resistance mechanisms specifically involving Akt and MAPK signalling branches in Figure 2A,C. Interestingly, a recent report assessing 943 neuroblastoma patients demonstrated the ALK R1275Q mutation is the most frequent at relapse, occurring *de novo* in the absence of *MYCN* amplification [115].

### 4.2 Role of ALK in metabolism – implications for neuroblastoma

Despite signalling to kinases implicated in major metabolic pathways, there is a paucity of information linking ALK to the regulation of cancer metabolism and, by extension, neuroblastoma. That loss of ALK protected against diet-induced obesity highlighted its ability to restrain lipolysis and maintain adipose tissue storage by controlling sympathetic tone (norepinephrine output) [83]. Whether ALK has an effect on lipid metabolism in neuroblastoma *in vivo*, however, remains unknown, but is not entirely implausible when contextualising its downstream effectors. Akt has a defined role in driving lipogenesis in an mTORC1-dependent and -independent manner that involves upregulation of sterol regulatory element binding protein 1 (SREBP1) [116], a key lipogenic transcription factor. While Akt-dependent lipogenesis is featured in the progression of a number of different cancers (e.g., liver, ovarian, prostate) [117], it is yet to be recognised as a driver of neuroblastoma. Regardless, in neuroblastoma cell lines in which *ALK* is either amplified (SK-N-AS: *ALK* is transcriptionally controlled by MYCN [90]) or mutated (LAN-5: ALK^R1275Q^ [118]), MYCN was reported to disrupt the normal cellular circadian rhythm to promote a lipogenic transcriptional program [119]. Taking advantage of RNA-seq, reactome pathway enrichment analysis revealed the most sensitive process to this MYCN-mediated disruption of the biological clock was “cholesterol biosynthesis and regulation by SREBP” [119].

The ALK interactome analysis (which flagged IRS2 as a major component of ALK signalling in neuroblastoma) discovered a number of glycolytic enzymes (glucose-6-phosphate isomerase, aldolase, triosephosphate isomerase 1, and phosphoglycerate mutase 1) in the ALK-interacting network [100]. These findings were supported by phosphoproteomics data demonstrating an ALK-regulated phosphorylation of AS160 (Akt substrate of 160 kDa) that controls GLUT4 trafficking and glucose uptake. These data also identified an ALK-sensitive phosphorylation site, S831, on the lipogenic enzyme fatty acid synthase (FASN), positioned in a linker connecting two FASN domains implicated in catalysis [120]. Immediately C-terminal to the phospho-site is a proline, indicating its preferencing by a Pro-directed kinase, of which many are involved in cellular growth and proliferation (e.g., mTORC1, ERK1/2, cyclin-dependent kinases (CDKs), etc.). Given their positive feedback [91], there is a possibility that ALK/MYCN cooperativity is involved in the oncogenic metabolic transformation (e.g., elevated glucose uptake/glycolysis and lipid synthesis) of neuroblastoma. Future work is therefore required to determine precise inputs (ligand-induced ALK activation and/or genetic alterations) and outputs (downstream signalling candidates and substrates) modulating this phenotype with a view to capitalise on any metabolic liabilities by therapeutic means. Surprisingly, ALK inhibition by lorlatinib, when administered as a single agent in neuroblastoma patients, caused weight gain and hypertriglyceridemia [110], an effect observed also in NSCLC patients [121], but not with other ALK inhibitors such as ceritinib [107]. Whether this change in lipid status is a direct consequence of ALK inhibition warrants further study. One might speculate it is a manifestation of defective IRS2 signalling to Akt and glucose uptake, which can precipitate metabolic derangements like elevated circulating lipids [122]. An overview of ALK signalling and its major, putative metabolic effects in neuroblastoma is provided in Figure 2C.

## 5 Provirus integration site for moloney murine leukemia virus (PIM) kinase

PIMs are Ser/Thr protein kinases encoded by three separate genes located at different chromosomes (*Pim1*, chr.6; *Pim2*, chr. X; *Pim3*, chr.22), giving rise to three isoforms (PIM1/2/3) displaying high-sequence conservation (61–71%) [123]. *Pim1*, the original isoform reported in the literature, was identified in the 1980s as an oncogene in mice with murine leukemia virus (MuLV)-induced lymphoma [124]. Due to multiple translation start sites, PIM1 and PIM2 are expressed as variants with differing molecular masses that localise to distinct cellular compartments.

**Figure 3:**
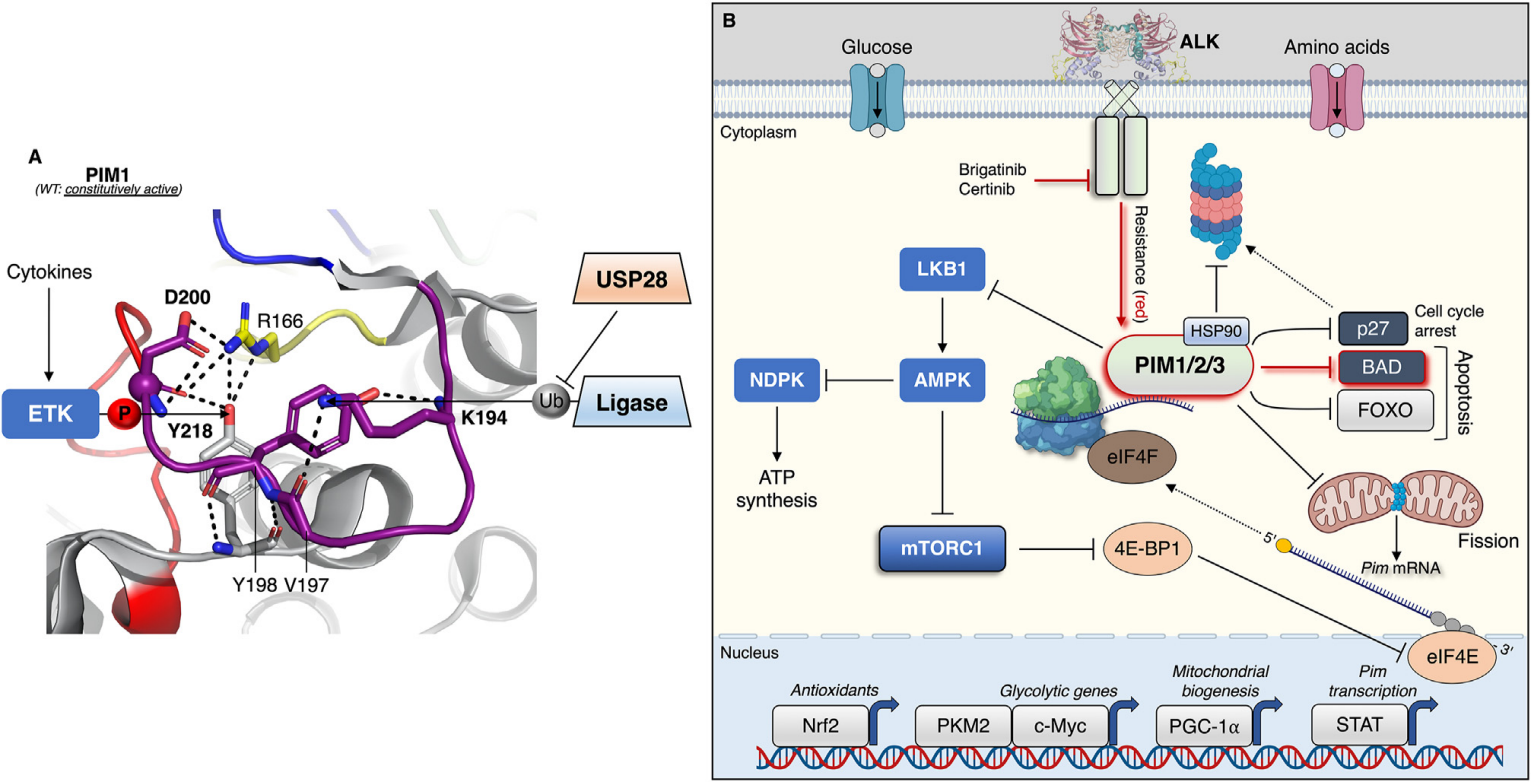
Regulation of PIM kinase activity and considerations for its role in neuroblastoma. **A**) PIM is synthesized as a constitutively active enzyme devoid of activation loop phosphorylation attributable to a phospho-mimetic D200 (numbering based on PIM1; PDB: 1XR1) at the canonical phospho-acceptor position. While phosphorylation is not absolutely required for activity, Y218, a substrate of ETK, albeit in response to cytokine stimulation, participates in the extensive hydrogen bonding network associated with the active enzyme. K194, a putative ubiquitination substrate, is also implicated in this network, and may affect protein stability via ubiquitin turnover (e.g., USP28-mediated deubiquitination). **B**) The global effects of the PIM kinase signalling milieu are an upregulation of metabolic processes that maximise energy extraction (e.g., PGC-1α-induced mitochondrial biogenesis), protection of the cells against oxidative stress (e.g., Nrf2-induced antioxidant defence) and progression through the cell cycle (inhibition of apoptotic mediators and p27). PIM expression is sensitive to nutrient status, probably through activation of the master growth regulator mTORC1, and as such may promote ATP synthesis via inhibition of AMPK. Like what was presented for ALK in Figure 3C, drug resistance implicating activation of PIM and downstream signalling is indicated in red.

Unlike the conventional mode of regulation of the majority of eukaryotic protein kinases (ePKs), PIM kinases are constitutively active due to several unique structural features, including a phospho-mimetic aspartate residue (D200; numbering based on PIM1) in place of the canonical activation loop phospho-acceptor site that interacts with a crucial arginine (R166 of the HRD motif described in Figure 1A) in the catalytic loop (Figure 3A) [125,126]. Noteworthy instances of upstream kinase regulation have been reported, such as ETK (epithelial and endothelial tyrosine kinase) that was shown to phosphorylate the PIM1 residue Y218 (situated in a loop between the APE motif and αF helix), elevating its activity in prostate cancer following cytokine (IL-6) treatment [127]. The sidechain hydroxyl group of Y218 also participates in the hydrogen bonding network with R166 and D200 (Figure 3A), indicating that phosphorylation here might affect some of these interactions. Regardless, PIM kinase activity is predominantly regulated at the level of transcription-translation and stability of the synthesized protein, as evidenced by stimulus-driven increases in PIM expression being proportionate to levels of its activity [128]. *Pim* transcripts have a short half-life and are intrinsically unstable as a consequence of five copies of AUUU(A) sequence motifs in the 3′ untranslated region (UTR) [129,130]. These sequences can be removed, for example, by proviral (MuLV) integration into the 3′ UTR region, leading to elevated protein abundance [131]. Moreover, the *Pim1* 5′ UTR contains a 400-nucleotide stretch comprising 76% GC content that confers inhibition toward translation. Removal of this sequence, or overexpression of the eukaryotic translation initiation factor (eIF) eIF4E, is sufficient to enhance *Pim1* gene expression [132], in which eIF4E relieves 5′ UTR-mediated inhibition of *Pim1* and promotes cap-dependent translation [132]. Notably, eIF4E is generally required for cap binding and presentation of a given mRNA transcript to the translation initiation/ribosome-recruiting heterotrimeric complex eIF4F. *Pim1* was found to contain a ∼50-nucleotide element in its 3′ UTR termed an “eIF4E sensitivity element,” which allows for association with eIF4E, triggering nuclear export and enhanced rates of translation [133]. Because *Pim2* is also regulated at the transcriptional level indicates overlapping mechanisms guiding the expression of each isoform [134]. *Pim* gene expression is rapid and induced in response to mitogenic stimuli such as interleukins and JAK/STAT signalling [123].

Like the mRNA, PIM proteins are also unstable and have a noticeably shorter half-life in normal peripheral blood leukocytes (<5 min) than in chronic myelogenous leukemia cells (<20 min) [135], suggesting posttranslational regulatory mechanisms controlling their stability. The Ser/Thr protein phosphatase PP2A has been shown to diminish PIM protein levels by facilitating its ubiquitin-mediated proteasomal disposal [136,137]. This implicates PIM1 interacting with heat shock proteins, whereby association with HSP90 and HSP70 prevents, and promotes, proteasomal degradation respectively [138,139]. Hypoxia-induced increases in PIM protein levels are transcription-independent [[140], [141], [142], [143]], and occur through deubiquitination of at least PIM1 and PIM2 by the deubiquitinase USP28 [143]. While activation loop phosphorylation is not required for PIM catalytic activity *per se*, the observation that PP2A phosphatase activity is involved in modulating its ubiquitination and degradation points to the likelihood of a stimulus-driven (e.g., hypoxia) phosphorylation cycle controlling PIM abundance. PIM1 and PIM3 activation loop lysines, 194 and 197 respectively, have been identified as ubiquitination sites in 12 high-throughput studies [144], raising speculation that their ubiquitination status is sensitive to a proximally positioned phosphorylated residue(s). Lysine charge neutralisation via covalent attachment of a ubiquitin moiety would disrupt several of the internal interactions that render PIM constitutively active, which would be protected by deubiquitination (Figure 3A).

The proto-oncogenic activity of PIM1 was initially confirmed in transgenic mice via its cooperation with c-Myc and MYCN, predisposing these mice to lymphomagenesis [145]. PIM was later shown to cooperate with other oncogenic factors (e.g., Ras) that propagate disease [146,147]. Since then, overexpression of PIM kinases has been described in a range of other cancers, not just haematological malignancies; for example, prostate adenocarcinoma, gastric carcinoma, colon carcinoma and hepatocellular carcinoma, often correlating with poor prognosis [123]. Mechanisms of action of PIM kinases in cancer involve phosphorylation and inactivation via proteasomal degradation of the cell cycle inhibitor p27, and phospho-inhibition of the FOXO1 and FOXO3 transcription factors, collectively inducing cell proliferation and survival [148].

### 5.1 Role of PIM kinases in neuroblastoma

In an analysis of two independent neuroblastoma patient cohorts, elevated *Pim* expression was associated with poorer overall survival [149,150]. Brunen and colleagues [150] demonstrated high-protein levels of PIM1 and PIM3 in 14 neuroblastoma cell lines, while Trigg and co-workers [149] assessed *Pim* mRNA across 25 neuroblastoma cell lines and found no correlation between isoform expression and *ALK* or *MYCN* status. Genetic disruption of *Pim1* expression was sufficient to reduce the viability of KELLY and SH-SY5Y cell lines [150]; however, in both studies, pharmacological inhibition of PIM (using pan-inhibitors AZD1208 and PIM-447) at clinically relevant doses yielded mixed efficacy in terms of preventing colony formation and reducing cell viability [149,150]. Nevertheless, taking advantage of a genome-wide CRISPR-Cas9 screen, Brunen et al. [150] pinpointed the absence of NF1 as a potential culprit causing resistance to PIM inhibition, which was confirmed *in vivo* as NF1-null xenografts were resistant to AZD1208 treatment. This is in keeping with reports of loss of NF1 function causing relapse in neuroblastoma [69,151], and the contribution of *NF1* mutations to ALK inhibitor resistance [112]. It should be pointed out however, that these genetic events are rare (<10%) when compared with the frequency of direct alterations to the Ras/MAPK pathway in relapsed neuroblastoma [69].

Genome-wide CRISPR activation screens of neuroblastoma cells treated with ALK inhibitors brigatinib or ceritinib for 14 days identified *Pim1* as a resistance gene, due to an anti-apoptotic phosphorylation of BAD by PIM1 [149]. PIM1 overexpression and knockdown experiments caused resistance and sensitization to pharmacological ALK inhibition, respectively. In patient-derived xenografts harbouring the common activating ALK mutations (F1174L, F1245C), combinatorial ALK (ceritinib) and PIM1 (AZD1208) inhibition displayed greater anti-tumour efficacy than either agent in isolation [149]. Such a result could hold promise in the clinic, since PIM inhibitors are fairly well-tolerated in humans [152]. Recently, the anti-tumour effects of the triple PIM/PI3K/mTOR kinase inhibitor IBL-302 was interrogated following preliminary screening of hundreds of cell lines from ∼50 different tumours that revealed neuroblastoma was especially sensitive to triple kinase inhibition [153]. Here, low-dose IBL-302 (50% of the maximum tolerated dose) and conventional chemotherapy (cisplatin) exacerbated the disruption of neuroblastoma growth in a patient-derived xenograft model [153]. IBL-302 also diminished MYCN expression in neuroblastoma cells, but whether this is attributable to a loss of PIM activity is unknown. It is worth mentioning however, that *PIM3* expression is higher in *MYCN*-amplified versus non-amplified tumours [150], suggesting possible isoform-specific roles depending on disease stage/genetic characteristics.

### 5.2 Role of PIM kinases in metabolism – implications for neuroblastoma

In 2011, Beharry and colleagues [154] demonstrated that genetic loss of all three *PIM* isoforms in mouse embryonic fibroblast (MEF) cells lowered the cellular energy charge, activating the energy-sensing AMP-activated protein kinase (AMPK), which inhibits mTORC1 and stunts cell growth [154]. These effects could be reversed by ectopic expression of PIM3 that lead to increased levels of the master regulator of mitochondrial biogenesis, PGC-1α, to restore energy balance [154]. All three PIMs were later shown to phosphorylate and inhibit the upstream activating kinase for AMPK, liver kinase B1 (LKB1) on S334, relieving AMPK-mediated cellular growth arrest in breast (MCF7) and prostate cancer (PC3) cell lines [155]. These findings are pertinent as AMPK phosphorylates NDPK-A on S120, inhibiting its ability to synthesize ATP [156]. As touched upon earlier, the NDPK-A S120G mutation is a metastasis-promoting event in neuroblastoma [27,28], suggesting PIM inhibition and activation of AMPK would delay tumour growth and/or invasiveness. There are no studies to our knowledge directly addressing the metabolic regulatory role(s) of PIM kinases in neuroblastoma, thus we can only draw parallels from the modulation of bioenergetics by PIM kinases in other cancers/divergent cell lines.

Several reports have demonstrated that PIM is a regulator of glycolysis via its ability to enhance glycolytic enzyme expression and supply of pathway intermediates, diverting metabolism away from mitochondrial respiration [[157], [158], [159]]. For example, PIM2 facilitates energy production from glycolysis in colorectal cancer cells [160]. Two noteworthy findings from the latter study were 1) PIM2 expression is nutrient-dependent, and 2) loss of mTORC1 activity by rapamycin abolished PIM2-induced glycolysis [160], suggesting crosstalk between the two kinases in governing glycolytic metabolism. This hypothesis is not entirely unreasonable since eIF4E, which controls PIM expression, is activated by mTORC1. In ovarian cancer cells, PIM1 controlled glycolytic gene expression in a manner involving c-Myc [159]. In addition, PIM2, but not PIM1 or PIM3, has been shown to directly interact with and phosphorylate pyruvate kinase M2 (PKM2) on T454, leading to increased nuclear activity of PKM2 where it functions as a transcriptional coactivator that promotes glycolysis required for cell survival and proliferation (e.g., in lung cancer A549 cells) [161]. Moreover, MEF cells with a *PIM1/2/3*-null background expressing constitutively active K-Ras (G12V), succumb to apoptosis due to an inability to quench ROS [157]. This probably implicates, among other processes, PIM's enhancement of nuclear factor-erythroid 2 p45-related factor 2 (Nrf2), which controls the transcription of endogenous antioxidants [142].

Another point to consider is that PIM1 has been shown to repress Drp1, either by limiting its expression and/or blocking compartmentalisation to mitochondria [162,163]. The general output is a survival advantage by countering excessive fission-induced ROS emissions and maximising ATP production. In fact, in SH-SY5Y neuroblastoma cells, post-transcriptional upregulation of *Drp1* potentiates mitochondrial dysfunction arising from aberrant mitochondrial fission, culminating in cell death [164]. That Drp1 is involved in the survival of high-stage, glycolytic neuroblastoma cells seems paradoxical, and could suggest that PIM facilitates cell survival in this setting by fine-tuning Drp1 expression. In support of this assertion, mitochondrial fission can induce PIM1 expression and trigger glycolytic metabolism in human small airway epithelial cells [165], similar to the effects of Survivin in aggressive neuroblastoma cells [42]. PIM regulation, signalling, and its role in metabolism is conveyed schematically in Figure 3B. We acknowledge this illustration is a general and simplified overview of PIM signalling, and that many of these PIM-mediated cellular effects are presumably stimulus-specific and vary between isoforms.

## 6 Aurora kinase

The Auroras are a family of Ser/Thr protein kinases best known for their role in mitosis [166]. Aurora kinases are well-conserved across evolution, whereby budding and fission yeast express only a single Aurora kinase (Ipl1 and Ark1 respectively) as opposed to the three identified in mammalian cells, Aurora-A, -B and –C (57–75% sequence homology) [167,168]. As Aurora-A and -B are the most well-characterised, we will limit our discussion to these two. Despite being grouped phylogenetically into their own family of ePKs, the Auroras are closely related to the AGC (protein kinase A/G/C) family of protein kinases [169]. However, unlike AGC kinases which contain a C-terminal hydrophobic regulatory tail/extension required for activation, Aurora-A and -B activation is accommodated by protein–protein interactions [170].

**Figure 4:**
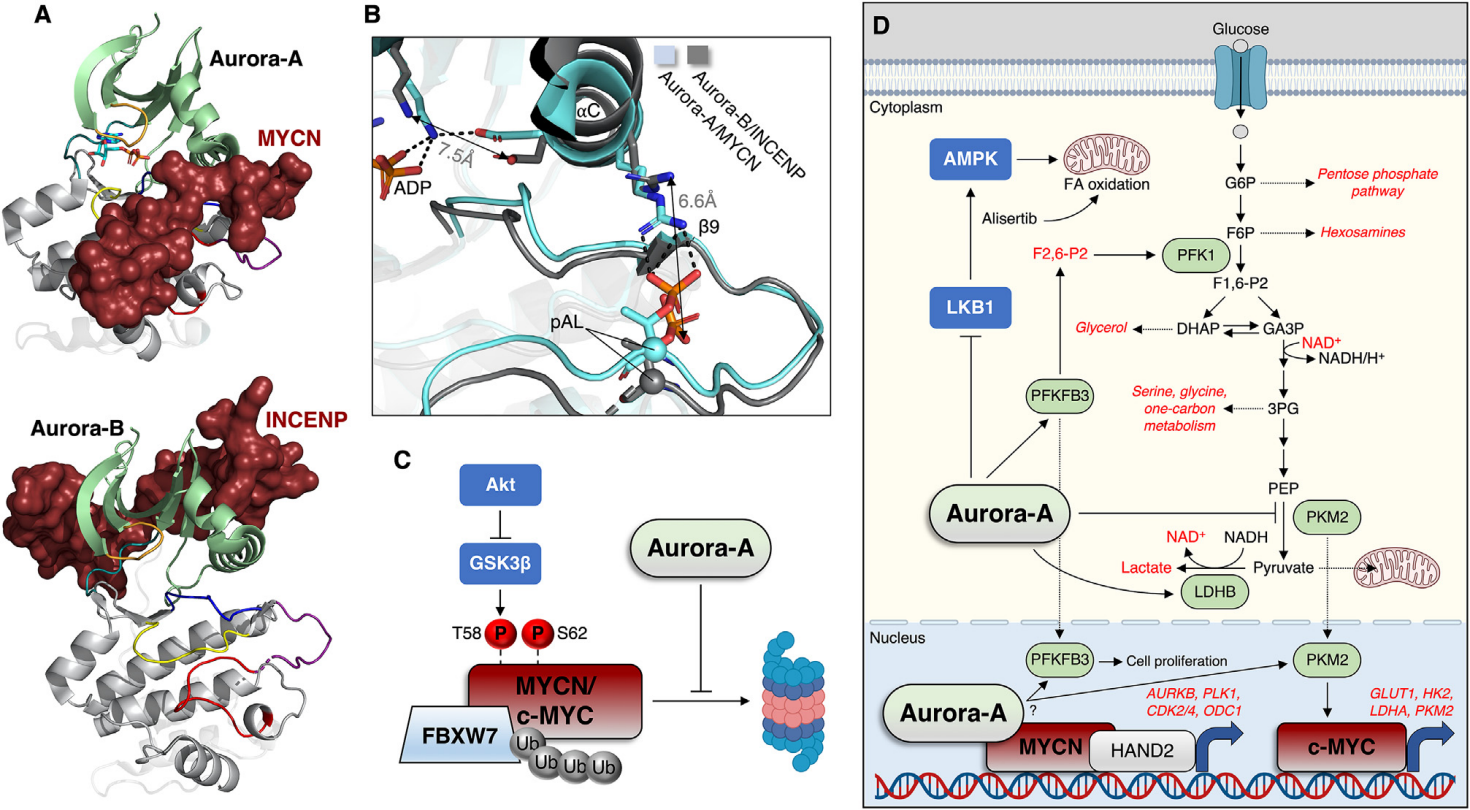
Regulation of Aurora-A and -B and control of the Warburg effect. **A**) Despite high-sequence similarity, Aurora-A and -B are associated with, and activated by, distinct binding partners, MYCN (PDB: 1OL5) and INCENP (PDB: 2BFX). **B**) Superimposition of the two kinases reveals that MYCN promotes a completely active configuration of Aurora-A, as demonstrated by the some of the structural trademarks described in Figure 1C, as well as a pAL/αC helix interaction. Conversely, INCENP promotes an intermediate state of Aurora-B activation, in which inward rotation of the αC helix physically separates relevant sidechains that typically bond within ∼3 Å (Å). **C**) As described in the text, both c-MYC and MYCN are ubiquitinated and degraded as a result of GSK3β phosphorylation under Akt-inhibited conditions, which can be overcome by Aurora-A binding to MYCN, and in the case c-MYC, Aurora-A inhibiting GSK3β phosphorylation by an unknown mechanism. **D**) Aurora-A regulates the Warburg effect that enhances cell proliferation (dependent on the utilisation of biosynthetic intermediates) by direct substrate phosphorylation and transcriptional activation through c-MYC. Aurora-A also transcriptionally regulates Aurora-B and other mitotic kinases via its interaction with MYCN, as well as *ODC1*, a vital enzyme in polyamine biosynthesis overexpressed in *MYCN*-amplified neuroblastoma.

Aurora-A maps to chromosome 20q13, a region frequently amplified in breast cancer that associates with poor prognosis [171]. Aurora-A has subsequently been found to be overexpressed in a variety of other cancers, for example pancreatic, ovarian and gastric cancer [167,172]. Aurora-A is required for chromosome segregation and genomic stability, as well as mitotic entry and spindle assembly [173], processes thought to explain its oncogenic potency. Aurora-A localisation to the mitotic spindle apparatus requires direct association with the motor-binding protein TPX2 that induces its activation by protecting Aurora-A activation loop T288 autophosphorylation against phosphatase pressure [174,175]. MYCN has also been shown to directly bind the Aurora-A kinase domain, associating with the active site cleft, activation segment and αG helix [176]. This interaction generates a fully active kinase (Figure 4A) reminiscent of active AGC protein kinases (e.g., PKA presented in Figure 1A) and the Aurora-A/TPX2 complex [175,176].

Aurora-B was originally identified in a polymerase chain reaction screen for kinases overexpressed in human tumours [177]. Aurora-B is the catalytic member of the chromosomal passenger complex (CPC) that consists of the inner centromere protein (INCENP), Survivin and Borelain, which forms during cell division and is required for mitotic progression, such as regulation of kinetochore-microtubule attachments, spindle assembly checkpoint control, and cytokinesis to name a few [178]. The structure of the INCENP-bound Aurora-B is shown in Figure 4B. Activation of the CPC is an elaborate process characterised by complex interplay with other mitotic kinases (summarised in [179]) whose relevance to neuroblastoma will be acknowledged later. Unlike MYCN that fully activates Aurora-A, INCENP allosterically induces an intermediate state of activation, defined by an extended, *trans*-autophosphorylated (T232) activation loop and open active site cleft, whereas full activation is achieved once Aurora-B phosphorylates C-terminal residues of INCENP [180]. Superimposition of the two Aurora kinases reveals that in the semi-active state, an INCENP-induced rotation of the Aurora-B αC helix prevents key interactions within the fully active enzyme that can be seen in the Aurora-A/MYCN complex (Figure 4B). Aurora-B phosphorylates S10 of H3, a marker of mitotic chromosomes thought to cause chromosome compaction [181,182].

Substituting only glycine 198 in Aurora-A, which determines its affinity for TPX2, to the corresponding asparagine 142 in Aurora-B, converts Aurora-A into a binding partner for INCENP where it then assembles into the CPC and functionally substitutes for Aurora-B in the cell cycle [183]. MYCN on the other hand attaches to distinct locations on the Aurora-A kinase domain. Ultimately, this unequivocally demonstrates that the physiological effects of the Aurora kinases are dictated primarily by their binding clientele, and the discovery and characterisation of other activatory proteins may yet broaden the scope of Aurora kinase biology [184,185]. In addition, Protein Kinase C epsilon (PKCε) has been shown to phosphorylate a neighbouring activation loop S227 site in Aurora-B that induces a switch in substrate selectivity and allows for cytokinesis abscission checkpoint exit (ensuring fidelity of chromosome segregation and completion of cell division) and DNA catenation resolution [186,187], suggesting other Aurora regulatory kinases may exist to dictate spatial and temporal activities.

### 6.1 Role of aurora kinase a and B in neuroblastoma

In 2009, Aurora-A was demonstrated to be overexpressed (mRNA and protein levels) in human neuroblastoma primary tissue and cell lines, which associated with clinically aggressive disease, tumour size and risk of relapse [188]. Otto and colleagues [189] found that Aurora-A was required for the growth of *MYCN*-amplified neuroblastoma cells. Specifically, Aurora-A could bind and stabilise the MYCN protein [189], which ordinarily undergoes degradation in response to a loss of growth factor PI3K/Akt signalling [62]. Degradation of MYC proteins commences with sequential phosphorylation of the MYC Box I region, firstly by a ‘priming’ phosphorylation on S62, followed by GSK3β phosphorylation of T58 [190,191]. MYC phosphorylated on these sites is consequently converted into a substrate of the ubiquitin ligase FBXW7 that targets it for proteasomal removal (Figure 4C) [189,191,192]. This process would usually allow for progression through mitosis and differentiation of neuronal progenitors [190], however overexpression of Aurora-A in neuroblastoma interrupts the normal mitotic regime. Aurora-A was shown to occupy the same binding region on MYCN as FBXW7, shielding MYCN from degradation [176], explaining why compounds that distort the Aurora-A kinase domain, untethering the Aurora-A/MYCN complex, also trigger MYCN destabilisation [176,193] and cause tumour regression *in vivo* in *MYCN*-driven neuroblastoma [194]. A first-in-class compound, HLB-0532259, has been developed that induces degradation of both Aurora-A and MYCN with high-selectivity and nanomolar potency in *MYCN*-amplified neuroblastoma cells [195]. Mechanistically, MYCN activates Aurora-A on the chromatin of neuroblastoma cells, where it phosphorylates S10 of histone H3 during the DNA-replicating S-phase of the cell cycle [196]. This contrasts with the chromatin enrichment of TPX2 and Aurora-A that takes place during the later G2/M phase of the cell cycle and transitions into mitosis. Briefly, TPX2 is known to be oncogenic in neuroblastoma [197,198], hence Aurora-A's oncogenicity is not likely restricted to MYCN binding. Association with Aurora-A limits MYCN-dependent transcription elongation to coordinate it with DNA replication [199]. Inhibition of Aurora-A during this phase causes transcription-replication conflicts that activate the ataxia telangiectasia and Rad3-related (ATR) kinase [196], an inhibitor of DNA double-strand breaks that upholds cell viability. In pre-clinical models of neuroblastoma, inhibiting both Aurora-A and ATR causes massive tumour apoptosis and disease eradication [196]. Finally, the interaction of MYCN with the transcription factor HAND2 (heart and neural crest derivatives-expressed protein 2), that permits MYCN chromosome accessibility and enhancer binding in neuroblastoma, was abolished by the Aurora-A inhibitor alisertib, disrupting tumour growth [200]. Direct targeting of Aurora-A illuminates an exciting new venture to combat the undruggablity of MYCN, particularly in high-risk neuroblastoma. One such avenue could involve use of PROTACs (Proteolysis Targeting Chimeras) comprising two chemical moieties that bind the kinase of interest and an E3-ubiquitin ligase, resulting in degradation of the target, in this case Aurora-A [201]. PROTACs elicited Aurora-A degradation at low nanomolar potency in leukemic cells and should now be considered as a therapeutic option in *MYCN*-amplified neuroblastoma [202].

Comparatively much less is known about the function of Aurora-B in neuroblastoma. In 2010, Aurora-B was implicated as a potentially druggable target for the treatment of neuroblastoma [203]. Bogen et al. [204] used a combined, unbiased approach of high-throughput RNAi and small molecule drug screens (465 compounds, four cell lines) to pinpoint Aurora-B as an actionable target in neuroblastoma. In that study, the Aurora-B-selective compound barasertib reduced the viability of *MYCN*-amplified, *TP53*-wild-type neuroblastoma cell lines at low nanomolar potency, as well as causing tumour regression *in vivo*, similarly in *MYCN*-amplified/*TP53*-wild-type neuroblastoma xenografts [204]. One could argue the tumourigenic impact of the Aurora-A and -B kinases is exacerbated by *MYCN*-amplification, an assertion supported by work using the dual Aurora-A/B kinase inhibitor CCT137690 that solely demonstrated efficacy in *MYCN*-amplified neuroblastoma cell lines and *in vivo* [205]. The oncogenic effects of Aurora-B in neuroblastoma are probably accounted for by its catalytic function in the CPC. To underscore this point, Polo-like kinase 1 (PLK1) and the monopolar spindle 1 (Mps1) kinase, two kinases involved in the regulation of the CPC, are therapeutic targets in high-risk neuroblastoma [206,207]. CPC disruption by targeting either INCENP or Survivin, whose expression correlates with high-risk disease in primary tumours, causes apoptosis in neuroblastoma cells [[208], [209], [210]], although at least for Survivin, it is unclear whether this is attributable to its part played in glycolytic metabolism [42]. Given the indispensable function of INCENP in activating Aurora-B (and by extension the CPC) from an intermediate to fully competent enzyme, strategies aimed at disrupting this component may be the most prudent means of alienating Aurora-B from the CPC and thus limiting its oncogenic function [208]. One final mention is the role played by PKCε in ensuring Aurora-B correctly finalises cell division, which is consistent with studies highlighting PKCε drives differentiation and migration of neuroblastoma cells [211,212].

Pathway crosstalk is evidenced by knockdown of MYCN and HAND2 in neuroblastoma cells (whose interaction as previously mentioned is regulated by Aurora-A) attenuating expression of the genes encoding Aurora-B and PLK1, amongst others [200]. Albeit in gastric cancer, Aurora-A has been shown to ensure the stability of Survivin by blocking the expression of a ubiquitin ligase that destines it for the proteasome [213].

### 6.2 Role of aurora kinase a in metabolism – implications for neuroblastoma

Of the Aurora kinases, several independent studies have shed light on the function of Aurora-A in the modulation of cancer metabolism, which we will focus on here. In glioblastoma, genetic and pharmacological (alisertib) inhibition of Aurora-A reversed the Warburg effect [214]. This was attributable to loss of c-MYC expression, coinciding with upregulated mitochondrial fatty acid oxidation; dual abrogation of Aurora-A and fatty acid oxidation prolonged the survival of mice in patient-derived xenograft models of glioblastoma [214]. Interestingly, kinase-active, but not kinase-dead, Aurora-A, stabilised c-MYC by preventing its phosphorylation by GSK3β and proteasomal targeting [214], which at first glance raises the possibility that Aurora-A binds to, and is also reciprocally activated by c-MYC. However, the Aurora-A interaction domain of MYCN is not conserved in c-MYC, hence further examination of this proposition is warranted. Direct control over glycolysis has been reported for Aurora-A. Aurora-A can phosphorylate lactate dehydrogenase B (LDHB) on S162 to relieve substrate inhibition by pyruvate and enhance the latter's conversion into lactate, regenerating NAD^+^ and driving glycolytic flux required for biosynthesis and tumour progression [215]. Expression of *Aurora-A* and *LDHB* are coincidently upregulated in human colon, lung and cervical cancer specimens [215], pointing to a possible general regulatory mechanism in divergent malignancies. Just recently, Aurora-A was shown to phosphorylate the multifunctional enzyme 6-phosphofructo-2-kinase/fructose-2,6-bisphosphatase, PFKFB3, on S461, which generates fructose 2,6-bisphosphate, an allosteric activator of 6-phosphofructo-1-kinase (PFK1) and rate-limiting enzyme in glycolysis [216], causing thyroid cancer progression. Aurora-A also shares substrates with PIM yet phosphorylates distinct residues. Firstly, T45 on PKM2 during the S-phase of the cell cycle, supporting lung cancer cell proliferation and tumour growth in mice [217]. This phosphorylation inhibits PKM2 by preventing its tetramerization, creating a bottleneck at the terminal stage of glycolysis and redirecting intermediates toward biosynthetic pathways (e.g., pentose phosphate pathway for nucleotide synthesis). Secondly, an inhibitory S299 phosphorylation of LKB1, causing diminution of AMPK activity in NSCLC and disease initiation and progression [218]. We suspect why Aurora-A and not Aurora-B features so heavily in controlling the Warburg effect is because of the greater representation of glycolytic enzymes during cell cycle phases preceding mitosis [219]. For example, in neuroblastoma, this would be coupled to the Aurora-A/MYCN modulation of DNA synthesis in S-phase [196]. Figure 4D gives as an illustration of how Aurora-A would theoretically drive these effects. One might suspect that glycolytic substrates of Aurora-A are cytoplasmic, namely LDHB, which may necessitate a novel activation mechanism. However, some glycolytic enzymes periodically translocate to the nucleus during the cell cycle, most notably the Aurora-A targets PKM2, that when released from its tetrameric assembly upregulates c-MYC-mediated glycolytic gene expression [220], and PFKFB3, whose function in the nucleus promotes G1/S transition [221]. Whether chromatin-localised, MYCN-bound Aurora-A is involved in their activation is a topic for future studies.

**Figure 5:**
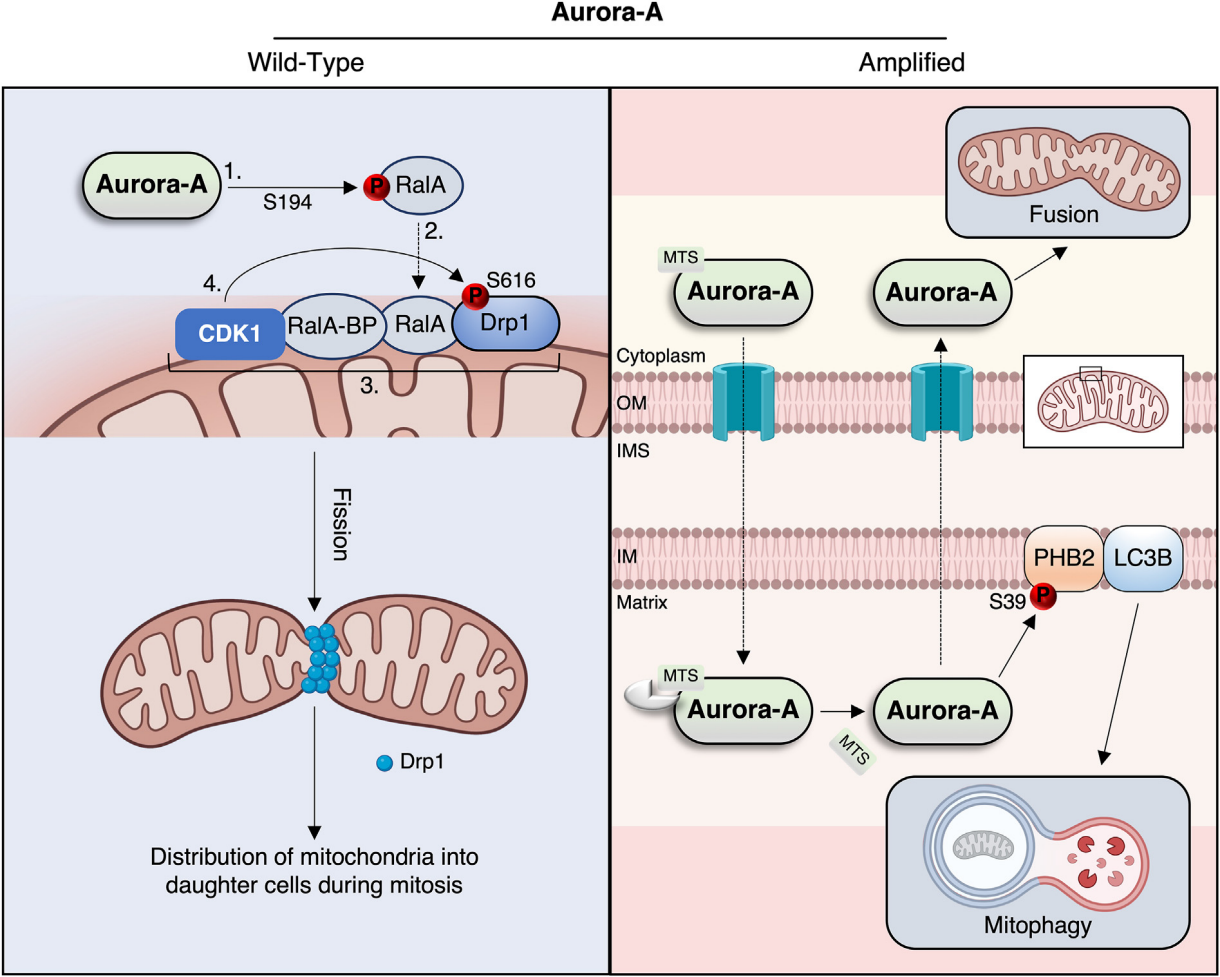
Divergent control of mitochondrial dynamics by Aurora-A. Left panel: wild-type Aurora-A promotes mitochondrial fission via phosphorylation of the RalA GTPase (1) that is scaffolded to mitochondria via its binding protein RalA-BP (2), thereby facilitating activating phosphorylation of Drp1 by CDK1 (3, 4); this process ensures proper distribution of mitochondria into dividing cells during mitosis. Right panel: amplified Aurora-A is thought to be imported into mitochondria via its N-terminal MTS (mitochondria targeting sequence), which is proteolytically cleaved upon infiltration of the matrix. Here, Aurora-A phosphorylates the mitophagy receptor prohibitin-2 (PHB2) to promote an interaction with LC3B and drive mitophagy and elimination of defective organelles. Aurora-A exported from the mitochondria promotes mitochondrial fusion via unknown mechanisms, and thus maximal ATP production through efficient OXPHOS.

A number of investigations have additionally elucidated Aurora-A's ability to control mitochondrial dynamics. Aurora-A contains an N-terminal mitochondria-targeting sequence that localises it to the mitochondrial matrix/inner membrane [222,223]. It is here where Aurora-A induces mitochondrial autophagy (removal of damaged organelles) and fusion (i.e., elongation) of the existing mitochondrial pool to boost ATP production [223,224]. However, these effects have been shown to be contingent upon overexpression of the kinase (pathway presented in greater detail in Figure 5). By contrast, under physiological conditions, mitotic mitochondrial fission during the earlier prophase implicates a CDK1-catalysed phosphorylation of the activating S616 site on Drp1 in a manner facilitated by Aurora-A (Figure 5) [225,226]. Regardless, it is unclear whether Aurora-A-induced mitochondrial autophagy/fusion cycles, when the kinase is overexpressed, is only relevant for the survival of cancerous cells (e.g., epithelial) that have a more oxidative phenotype [227]. Any mitotic stress events (centrosome abnormalities, chromosome misalignment, aberrant DNA inheritance) provoked by overexpression of Aurora-A [173,228], would be ameliorated by the anti-apoptotic effects of these mitochondrial quality control processes that optimise ATP production for cell survival. As we have pointed out, *MYCN*-amplification augments mitochondrial function and OXPHOS in neuroblastoma tumours [45,46,50], therefore targeting Aurora-A might reverse this phenomenon and expose metabolic vulnerabilities previously deemed evasive due to the so-called undruggablity of MYCN.

## 7 Metabolic and genetic heterogeneity of neuroblastoma presents a case for targeted therapies

The reliance of aggressive (i.e., *MYCN*-dependent) neuroblastoma on OXPHOS points at first glance to the use of mitochondrial inhibitors to circumvent treatment resistance. Because these inhibitors would also trigger AMPK activity as a result of an energy deficit, one would expect a canonical, AMPK-mediated inhibition of mTORC1 and cell cycle arrest to address the energy shortfalls needed for cell cycle progression [229]. Moreover, Akt and mTORC2 are indirectly and directly activated by AMPK, respectively, specifically in response to nutrient stress [230,231], which is consistent with this pathway executing cell survival functions. A precedent has already been set in melanoma-bearing mice; combined implementation of the OXPHOS inhibitor IACS-010759, and Atorvastatin, a hydroxymethylglutaryl-CoA reductase (HMGCR) and cholesterol synthesis inhibitor, completely abrogated tumour growth [232]. Dual therapies limited Akt/mTORC2 signalling despite persistent AMPK activity, providing evidence of a failed cell survival effort [232]. From these observations, it could also be speculated that highly specific inhibitors of ALK, PIM or Aurora-A/B (depending on the genetic characteristics and stage of the tumour) alongside metabolic inhibitors would markedly disrupt the progression of neuroblastomas whose aggressiveness is potentiated by the actions of these signalling pathways, particularly when in combination with *MYCN*-amplification. Hence the idea would be to achieve similar outcomes as dual Aurora-A and ATR inhibition and the profound effect that has on apoptosis of *MYCN*-amplified neuroblastoma tumours [196]. Furthermore, targeted inhibition of Aurora-A in high-risk neuroblastoma could emerge as a principal strategy aimed at breaking its highly oncogenic partnership with MYCN. While PIM and Aurora-A inhibition as a monotherapy would theoretically inhibit glycolysis and activate AMPK-mediated fatty acid oxidation, drug synergy approaches might override any cell survival efforts mounted by AMPK, as well as blunting mitochondrial adaptations that would otherwise accommodate for changes in nutrient status.

## 8 Conclusions

The complexity of neuroblastoma and efforts to treat this disease are complicated by the genetic and metabolic heterogeneity of the tumours. What is overwhelmingly apparent is that neuroblastomas deploy elaborate resistance mechanisms when exposed to currently approved pharmaceuticals that impact patient prognosis and future outcomes. Further complicating treatment approaches is the growing appreciation that neuroblastoma metabolism does not entirely conform to the classical glycolytic “Warburg effect,” and that high-risk tumours, in particular *MYCN*-amplified, have considerable metabolic flexibility and a capacity to harness mitochondrial OXPHOS for energy extraction. As dietary intervention is becoming an increasingly promising anti-cancer strategy [233], development of inhibitors with minimal off-target effects and toxicity profiles, in combination with the relative safety and feasibility of dietary interventions, such as the ketogenic diet that has shown promise in preclinical neuroblastoma models [36,234,235], could represent an exciting frontier for treatment in the field. Knowledge would also be immensely advanced by future, detailed metabolic characterisations of the kinases discussed herein, in particular PIM and Aurora-A, since presently the extent of our understanding is hampered by the vast majority of studies in this realm being conducted in cancers other than neuroblastoma.

## Author contributions

WJS and BK were involved in conceptualisation of the review. WJS wrote the original draft version and constructed all the figures. Each author contributed to editing and revisions and were responsible for the final content.

## FUNDING

Children’s Cancer Foundation, Salzburg, Austrian Science Fund: FWF P 31228-B33. PMU Research Promotion Fund: 2022-PRE-006-Catalano and A-18/02/032-KOF.

## Declaration of Competing Interest

The authors declare that they have no conflicts of interest.

## Data Availability

No data was used for the research described in the article.

## ABBREVIATIONS

4E-BP1: eIF4E-binding protein 1
3′ UTR: 3′ untranslated region
AGC: Protein kinase A/G/C family
ALK: Anaplastic lymphoma kinase
AMPK: AMP-activated protein kinase
AS160: Akt substrate of 160 kDa
ASCT2: Solute carrier family 1 member 5, SLC1A5
ATR: Ataxia telangiectasia and Rad3-related
BAD: Bcl-2-associated agonist of cell death
CDK: Cyclin-dependent protein kinase
CPC: Chromosomal passenger complex
CTE: C-terminal extension
c-SRC: Proto-oncogene tyrosine protein kinase, cellular sarcoma
DLST: Dihydrolipoamide S-succinyltransferase
Drp1: Dynamin-related protein 1
EGF: Epidermal growth factor
EGFR: Epidermal growth factor receptor
eIF: Eukaryotic translation initiation factor
ePK: Eukaryotic protein kinase
ERK1/2: Extracellular signal-regulated protein kinase ½
ETK: Epithelial and endothelial tyrosine kinase
ETV: E-twenty-six (ETS) domain-containing protein
FA: Fatty acid
FASN: Fatty acid synthase
FBXW7: F-box and WD repeat domain-containing 7
FOXO: Forkhead box transcription factor
GLUT4: Glucose transporter 4
GlyR: Glycine-rich region
Grb2: Growth factor receptor-bound protein 2
GSK3β: Glycogen synthase kinase 3β
HAND2: Heart and neural crest derivatives-expressed protein 2
HDAC: Histone deacetylase
HIF: Hypoxia-inducible factor
HMGCR: Hydroxymethylglutaryl-CoA reductase
HSP: Heat shock protein
INCENP: Inner centromere protein
IRS2: Insulin receptor substrate 2
JAK: Janus kinase
KD: Kinase domain
LDHB: Lactate dehydrogenase B
LC3B: Light chain 3B
LKB1: Liver kinase B1
LTK: Leukocyte tyrosine kinase
MAPK: Mitogen-activated protein kinase
MDM2: Mouse double minute 2
MEF: Mouse embryonic fibroblast
MEK1/2: Dual-specificity MAPK kinase 1/2
Mps1: Monopolor spindle 1
mtDNA: Mitochondrial DNA
mTORC: Mammalian target of rapamycin complex
MTS: Mitochondria targeting sequence
MuLV: Murine leukemia virus
NDPK-A: Nucleoside-diphosphate kinase-A
NF1: Neurofibromin
Nrf2: Nuclear factor-erythroid 2 p45-related factor 2
NSCLC: Non-small-cell lung cancer
NTE: N-terminal extension
ODC1: Ornithine decarboxylase 1
OXPHOS: Oxidative phosphorylation
PDB: Protein data bank
PDGF: Platelet-derived growth factor
PDGFR: Platelet-derived growth factor receptor
PDK1: Phosphoinositide-dependent protein kinase 1
PFK1: 6-phosphofructo-1-kinase
PFKFB3: 6-phosphofructo-2-kinase/fructose-2,6-bisphosphatase
PGC-1α: PPARγ-coactivator-1α
PHB2: Prohibitin-2
PI3K: Phosphatidylinositol 3-kinase
PIM: Provirus Integration Site for Moloney Murine Leukemia Virus
PIP_3_: Phosphatidylinositol-3,4,5-trisphosphate
PKA: Protein kinase A
PKCε: Protein kinase C epsilon
PKM2: Pyruvate kinase M2
PLCγ: Phospholipase Cγ
PLK1: Polo-like kinase 1
PP2A: Protein phosphatase 2A
PROTAC: Proteolysis Targeting Chimera
PTEN: Phosphatase and tensin homologue
RalA-BP: RalA-binding protein
ROS: Reactive oxygen species
RTK: Receptor tyrosine kinase
SETD8: SET domain-containing lysine methyltransferase 8
SOS: Son of sevenless
SREBP1: Sterol regulatory element binding protein 1
SRF: Serum response factor
STAT: Signal transducer and activator of transcription
TCA: Tricarboxylic acid
TENM3: Teneurin transmembrane protein 3
THB: Tri-helical bundle
TMH: Transmembrane helix
TNF: Tumour necrosis factor
TPX2: Targeting protein for Xklp2
USP28: Ubiquitin-specific peptidase 28
